# Fabrication of thermo-sensitive lignocellulose hydrogels with switchable hydrophilicity and hydrophobicity through an SIPN strategy

**DOI:** 10.1039/c9ra05575d

**Published:** 2019-09-18

**Authors:** Jianyu Xia, Zhulan Liu, Yan Chen, Zhiguo Wang, Yunfeng Cao

**Affiliations:** Jiangsu Provincial Key Lab of Pulp and Paper Science and Technology, Jiangsu Co-innovation Center of Efficient Processing and Utilization of Forest Resources, Nanjing Forestry University 159 Longpan Rd Nanjing 210037 China liuzhulan6202@sina.com yunfcao@163.com

## Abstract

Herein, thermo-sensitive lignocellulose hydrogels with varying lignin contents were fabricated with *N*-isopropylacrylamide (NIPAAm) by a semi-interpenetrating polymer network (SIPN) strategy using a LiCl/DMSO solvent system. Soda lignin mixed with the lignocellulose/LiCl/DMSO solution was also used to prepare the composite hydrogels, and the influence of the existential state of lignin on the hydrogel properties was analyzed objectively. The SIPN hydrogels exhibited more favorable mechanical properties due to the physical entanglement of poly-NIPAAm and lignocellulose. The presence of externally added lignin in the composite hydrogels is beneficial for mechanical improvement. Both the mechanical properties and the morphologies of the SIPN hydrogels can be tuned by varying the existential state and content of lignin. Furthermore, the prepared SIPN hydrogels showed rapid conversion from being hydrophilic at 20 °C to being hydrophobic at 45 °C. All SIPN hydrogels exhibited obvious oil absorbency in an oil/water mixture at 45 °C. Moreover, the different lignin existential states in the hydrogels resulted in different lower critical solution temperatures (LCST). This study provides a feasible route to produce reinforced thermo-sensitive hydrogels and develops a method for tailoring the morphology and the absorption properties of hydrogels by controlling the existential state and content of lignin.

## Abstract 2

This study provides a feasible route to produce reinforced thermo-sensitive hydrogels and tailor their morphologies and absorption properties by controlling the state and content of lignin.

## Graphical abstract

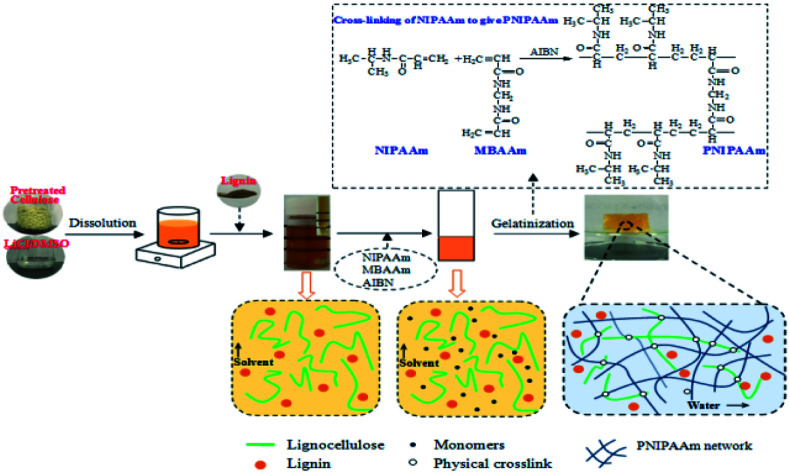

## Introduction

Hydrogels are cross-linked (physically or chemically) hydrophilic polymers composed of three-dimensional structures, and they can absorb large amounts of water and swell in water systems without dissolution.^1–5^ Recently, intelligent hydrogels, also known as stimuli-responsive hydrogels, have attracted significant attention due to their hydrophilicity, permeability, and response to environmental stimuli such as temperature,^6–8^ pH,^9–11^ light,^12^ ionic strength,^13^ magnetism,^14^ and electromagnetic radiation.^15^ Among these stimuli-responsive hydrogels, thermo-sensitive hydrogels are most widely used since temperature is an important environmental factor in different application systems. As a typical thermo-sensitive monomer, *N*-isopropylacrylamide (NIPAAm) has been widely used to synthesize thermo-sensitive hydrogels. When exposed to different external temperatures, these smart hydrogels can achieve hydrophilic–hydrophobic phase transitions around lower critical solution temperatures (LCST) due to the presence of hydrophobic isopropyl groups and hydrophilic amino groups in the network.^2,16^ They have been widely applied in biotechnology areas and wastewater treatment including oil/water separation. However, the applications of these hydrogels are limited by the lack of biodegradability and biocompatibility. Thus, it is necessary to incorporate natural polymers into these hydrogels to improve the environmental friendliness of these hydrogel systems.

As a representative of biomass, lignocellulose is an ideal candidate for the preparation of biocompatible materials due to its abundance and good renewability.^17,18^ Most researchers focus on the preparation and application of cellulose-based thermo-responsive hydrogels by choosing lignin-free cellulose materials as the feedstock for their mature technology.^19,20^ However, the achievement of cellulose from lignocellulose materials requires a series of harsh separation and purification processes, and the usage of bleaching chemicals would increase the cost and decrease the environmental compatibility of the biomass.^21^ Furthermore, lignin, the most abundant aromatic polymer in nature, cements the cellulose and hemicellulose together in lignocellulose, providing mechanical support and rigidity.^22,23^ In addition, the numerous functional groups of lignin, such as phenolic and aliphatic hydroxyl, methoxyl and carbonyl groups, endow it with sufficient reactivity with various compounds. Different studies have shown that incorporation of lignin in PVA hydrogels,^24^ epoxy resins^25^ and polyurethane products^26^ can effectively improve their mechanical properties and increase their renewable content. In addition, as a complex network of different phenylpropane units, lignin provides necessary hydrophobicity to plant cell walls. Polyester coatings and melamine sponges were functionalized with higher hydrophobic character by incorporating lignin into their systems.^27,28^ Thus, it is reasonable to hypothesize that introducing lignin can benefit the production of thermo-sensitive hydrogels, and the different existential states of lignin may also endow smart hydrogels with different properties.

Benefitting from the prior studies of the LiCl/DMSO solvent system in our group,^29–33^ ethylenediamine (EDA)-pretreated lignocellulose could be completely dissolved in LiCl/DMSO to afford a homogeneous solution under mild conditions (65 °C). Dissolution of lignocellulose in LiCl/DMSO requires pretreatment by either ball-milling or EDA complexation. Also, EDA-pretreatment can avoid mechanical degradation caused by ball-milling pretreatment.^34^ Furthermore, the subsequent dissolution after EDA-pretreatment does not require high temperatures; thus, structural modification of the lignin and cellulose degradation can also be avoided. The obtained lignocellulose solution can be used as the working medium for the fabrication of thermo-sensitive lignocellulose hydrogels.

In this study, lignocellulose-based thermo-sensitive hydrogels with varying lignin contents were fabricated from wheat straw pulp-LiCl/DMSO solution *via* the SIPN strategy. Cellulose/lignin composite thermo-sensitive hydrogels were also prepared by the external addition of lignin. The chemical structures, morphologies, mechanical properties and absorption capacities were characterized, and the influences of the content and existential state of lignin on these properties were analyzed in detail. Both the mechanical properties and the morphologies of SIPN hydrogels can be tuned by varying the existential state and content of lignin. Moreover, the thermo-sensitive performance and oil absorbency were also studied.

## Experimental

### Materials

Oxygen-delignified wheat straw soda pulps with lignin contents of 4.27% (LC-1), 3.25% (LC-2) and 2.26% (LC-3) were provided by a local pulp and paper-making factory (Quanlin Paper, Shandong, China). The lignin contents were determined by the method of NREL/TP-510-42618.^35^ These pulps were pretreated with ethylenediamine (EDA) with a concentration of 1 : 30 (solid–liquid ratio, m/v), subjected to freeze-drying, and then maintained in SEBC bottles. Soda lignin was acid-precipitated and purified from the black liquor of wheat straw soda pulping. *N*-isopropylacrylamide (NIPAAm) was a product of Energy Chemical (Shanghai, China). *N*,*N*-methylenebisacrylamide (MBAAm) was purchased from Chemical Reagent Institute (Tianjing, China). Azodiisobutyronitrile (AIBN) was purchased from Aladdin (Shanghai, China). LiCl and DMSO were of analytical-reagent grade and were used as received.

### Preparation of SIPN hydrogels

Lignocellulose SIPN hydrogels were prepared from wheat straw pulp according to the following procedure. Firstly, a lignocellulose solution was prepared according to our previous studies.^29–33^ EDA-pretreated pulp was added to 8% LiCl/DMSO solvent at room temperature with 2 wt% concentration. The suspension was continuously stirred for 24 h and then maintained at 65 °C for 5 h under stirring to obtain a homogeneous solution. After that, 1 g NIPAAm, 0.1 g MBAAm and 0.015 g AIBN were added to a tube containing 5 g wheat straw pulp-LiCl/DMSO solution with 2 wt% concentration. The mixture was stirred at room temperature until it was totally dissolved and then purged with nitrogen for 10 min. Then, it was sealed and placed in a water bath at 70 °C for 12 h to achieve gelatinization. The resulting gels were removed and washed with water for 5 days to remove the unreacted reagents, and the water was replaced every 12 h. The SIPN hydrogels prepared from LC-1, LC-2 and LC-3 were labeled as LCTG-1, LCTG-2 and LCTG-3, respectively.

For the cellulose/lignin composite thermo-sensitive hydrogels, different amounts of soda lignin were firstly added to 5 g LC-3/LiCl/DMSO solution, same as above, to reach the appropriate lignin contents in LC-1/LiCl/DMSO and LC-2/LiCl/DMSO. After dissolution, 1 g NIPAAm, 0.1 g MBAAm and 0.015 g AIBN were also dissolved in the solution. The mixture were then purged with nitrogen for 10 min and placed in a water bath at 70 °C for 12 h to achieve gelatinization. The obtained gels were washed with water to remove the unreacted reagents, and the water was changed every 12 h. The prepared composite hydrogels containing the same lignin contents as LCTG-1 or LCTG-2 were labeled as LCTGs-1 and LCTGs-2, respectively. The PNIPAAm hydrogel without lignocellulose was prepared from NIPAAm for comparison by the same procedure. The parameters for the synthesis of the thermo-sensitive hydrogels are shown in Table 1.

**Table 1:** The parameters for the synthesis of the thermo-sensitive hydrogels

Sample	Raw material (lignin content^a^)	NIPAAm (g)	MBAAm (g)	AIBN (g)
LCTG-1	LC-1 (4.27%), 0.100 g	1	0.1	0.015
LCTG-2	LC-2 (3.25%), 0.100 g	1	0.1	0.015
LCTG-3	LC-3 (2.26%), 0.100 g	1	0.1	0.015
LCTGs-1	LC-3 + soda lignin (4.19%), 0.102 g	1	0.1	0.015
LCTGs-2	LC-3 + soda lignin (3.22%), 0.101 g	1	0.1	0.015
PNIPAAm	—	1	0.1	0.015

a The lignin contents were calculated based on the raw materials in LiCl/DMSO before adding the monomers.

### Characterization of hydrogels

Fourier transform infrared (FT-IR) spectra of the freeze-dried hydrogels were measured by an FT-IR 650 FTIR spectrometer (Gangdong, China). 3 mg freeze-dried hydrogels were mixed with 200 mg KBr and ground into powder for FT-IR testing in the range of 4000 to 400 cm^−1^. Cross sections of the freeze-dried hydrogels were coated with gold and then observed by a Quanta 200 scanning electron microscope (SEM, FEI, USA). Nitrogen adsorption measurements were performed using a Quadrasorb-evo™ Gas Sorption Surface Area and Pore Size Analyzer (Quantachrome, USA). The BET surface areas (*S*_BET_) and BJH pore diameters (*d*_v_) of the freeze-dried hydrogels were determined by measuring the nitrogen adsorption–desorption isotherms at liquid nitrogen temperature; before the measurements, all freeze-dried hydrogels without any collapse were degassed at 60 °C for 24 h.

Differential scanning calorimetry (DSC) curves were recorded on a DSC 214 (NETZSCH, Germany); 5 to 10 mg of hydrogels were heated from 10 °C to 50 °C with a heating rate of 5 °C min^−1^ under 40 ml min^−1^ nitrogen flow. The rheological analysis of the hydrogels was performed using an RS6000 rheometer (HAAKER, Germany) with a P20 Ti L plate and a P20 Ti L cone. All samples were prepared with diameters equal to that of the P20 Ti L plate and a thickness of 4 mm. The specimens were subjected to strain sweep tests over the range of 0 to 10 000 Pa at ambient temperature with a frequency of 1 Hz. The storage modulus (*G*′) and loss modulus (*G*′′) of the hydrogels were recorded. Compression stress–strain curves of the hydrogels were obtained with an AG-X plus mechanical tester (Shimadzu, Japan) equipped with an SLBL 500 N load cell. Each specimen was cut into a cylinder sharp (20 mm in diameter, 8 mm in height), then vertically compressed with a speed of 1 mm min^−1^ at room temperature. The samples were measured in triplicate, and the compressive stress and strain were calculated from the measured forces and sample displacements based on the initial dimensions of the hydrogels. Static contact angles of the freeze-dried SIPN hydrogels were measured using a T200-Auto 3 Plus contact angle goniometer (BiolinScientific, Sweden) at 20 °C and 45 °C. A drop (4 μL) of deionized water was deposited on the surface of each sample for the contact angle measurements.

The gravimetric method was employed to measure the equilibrium reswelling ratios of the freeze-dried hydrogels at different temperatures ranging from 20 °C to 45 °C. The freeze-dried samples were soaked in deionized water for 24 h at each temperature, then picked up and weighed after removing excess water on the surface with moistened filter paper. The equilibrium reswelling ratio (*Q*_e_) was calculated as follows:

1 Equilibrium reswelling ratio (*Q*_e_) = (*w*_s_ − *w*_d_)/*w*_d_

where *w*_s_ is the weight of the equilibrated swollen gel and *w*_d_ is the weight of the measured freeze-dried hydrogel sample.

The absorption kinetic curve at 20 °C was determined by the absorption capacity of water at a certain time interval.

Simultaneously, the oil absorbencies of the freeze-dried SIPN hydrogels were measured gravimetrically at a temperature of 45 °C. All dried samples were immersed in a soybean oil/water mixture (1 : 4, v/v) for 24 h; then, they were weighed after removing the excess oil, and the oil absorbency of soybean oil was calculated as follows:

2 Oil absorbency = (*w*_o_ − *w*_d_)/*w*_d_

where *w*_d_ and *w*_o_ are the weights of the freeze-dried hydrogel before and after oil absorption, respectively.

## Results and discussion

### Formation of SIPN hydrogels

**Fig. 1:**
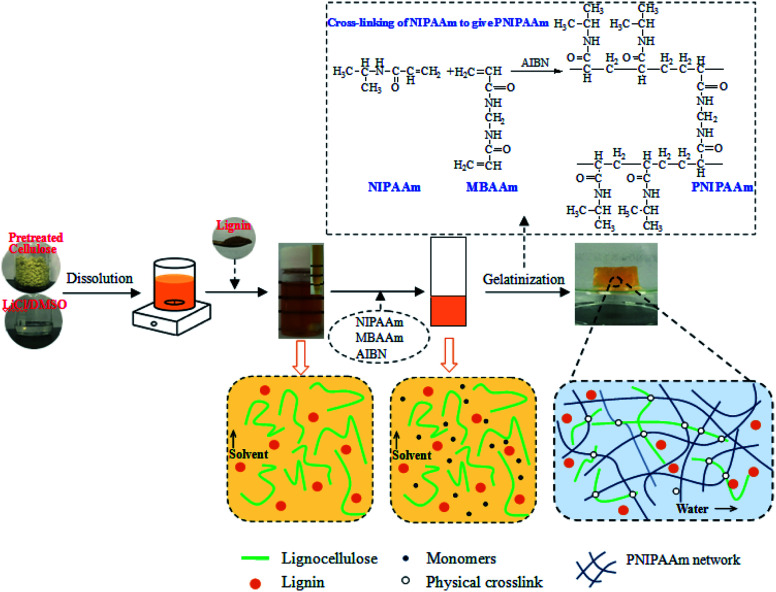
Schematic of the preparation of lignocellulose or composite hydrogels through the SIPN strategy.

The lignocellulose or composite thermo-sensitive hydrogels were prepared according to the schematic in Fig. 1 through the SIPN strategy. An interpenetrating polymer network (IPN) is a combination of two or more polymers in a network. For cellulose-based hydrogels, if the cellulose is only linear or branched in the other polymerized network, the gel is a semi-IPN hydrogel.^36,37^ In this study, the first PNIPAAm network was formed by the polymerization of NIPAAm with MBAAm as the cross-linker and AIBN as the initiator. Meanwhile, the lignocellulose existed as rigid rods and spanned throughout the PNIPAAm network to form SIPN thermally sensitive hydrogels, in which lignocellulose has the capacity to form hydrogen bonds between lignocellulose chains.^20^ For the cellulose/lignin composite hydrogels, soda lignin was firstly dissolved in LiCl/DMSO and dispersed in the gel network with solvent. Then, it was immersed in deionized water to regenerate as particles that were dispersed in the SIPN network due to the formation of intermolecular hydrogen bonds and van der Waals forces among lignocellulose, PNIPAAm and soda lignin. Moreover, the entanglements of the lignocellulose fibrils and the PNIPAAm network could embrace the lignin particles in the network.

### FTIR analysis of the SIPN hydrogels

**Fig. 2:**
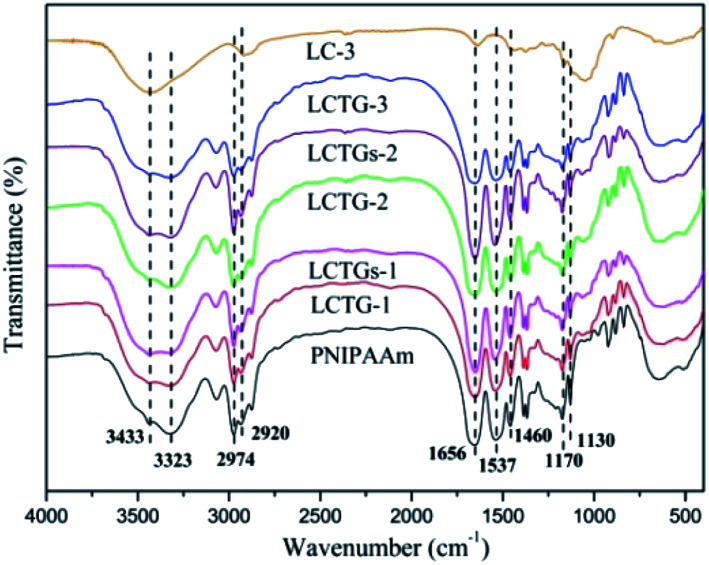
FTIR spectra of lignocellulose (LC-3), SIPN hydrogels and PNIPAAm hydrogel.

The FT-IR spectra of the lignocellulose material (LC-3), SIPN hydrogels and PNIPAAm hydrogel are shown in Fig. 2. The absorption peak at 3433 cm^−1^ is a characteristic band of hydroxyl groups. The broad absorption peak at 3323 cm^−1^ is attributed to the stretching vibration of amino groups.^38^ The characteristic absorption peaks at 2974 cm^−1^ and 1460 cm^−1^ correspond to the asymmetric stretching vibration and asymmetric deformation vibration of methyl groups, respectively.^6^ The characteristic absorption peak at 2920 cm^−1^ is attributed to the stretching vibration of methylene groups. The bands at 1170 and 1130 cm^−1^ are due to the existence of isopropyl groups, and the bands at 1656 and 1537 cm^−1^ are assigned to the typical amide group of NIPAAm.^20^ The presence of the amide groups and hydroxyl groups in the SIPN hydrogels proved that SIPN hydrogels composed of lignocellulose and PNIPAAm were successfully synthesized.

### Morphologies of the SIPN hydrogels

**Fig. 3:**
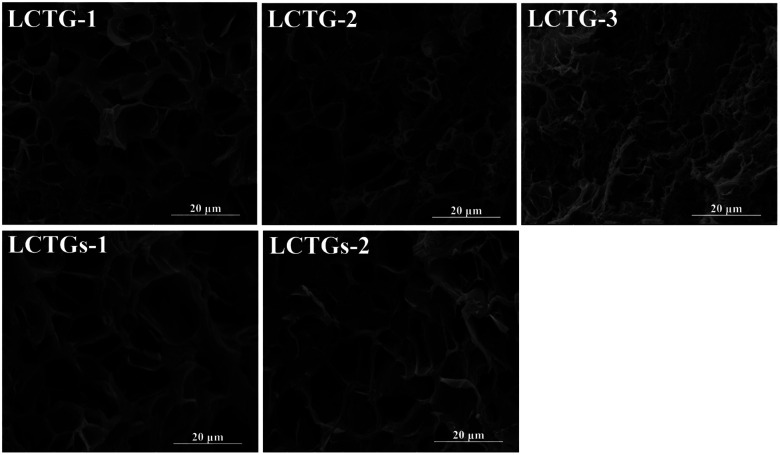
SEM images of cross-sections of the SIPN hydrogels.

SEM images of the cross sections of the SIPN hydrogels are shown in Fig. 3. It can be clearly observed that there are large amounts of pores inside the samples, which can endow the hydrogels with good absorption capacity. For LCTG-1, the pore size was much larger than those of LCTG-2 and LCTG-3. This can be attributed to the native lignin, which restricted the dissociation of cellulose and hemicellulose chains in the raw lignocellulose LC-1. Because the lignocellulose chains in the SIPN hydrogels existed as rigid rods throughout the PNIPAAm network, the aggregation of these three components, namely lignin, hemicellulose and cellulose, would intensively restrict the reaction between the monomer and the crosslinker, which resulted in a decrement of the cross-linking ratio and an increment of the pore size. Meanwhile, in LC-2 and LC-3, certain amounts of native lignin were removed by oxygen delignification, and the cellulose and hemicellulose could dissociate gradually.

Then, the restriction between the monomer and the crosslinker was decreased, and the interlacing of PNIPAAm and lignocellulose was increased. Thus, LCTG-2 and LCTG-3 could be obtained with denser porous morphologies. For LCTGs-1 and LCTGs-2, the dissolved lignin in LiCl/DMSO could also restrict the reaction between the monomer and the crosslinker, which accordingly induced much larger pores in the hydrogel network compared with that of LCTG-3.

### Nitrogen adsorption analysis of the hydrogels

**Fig. 4:**
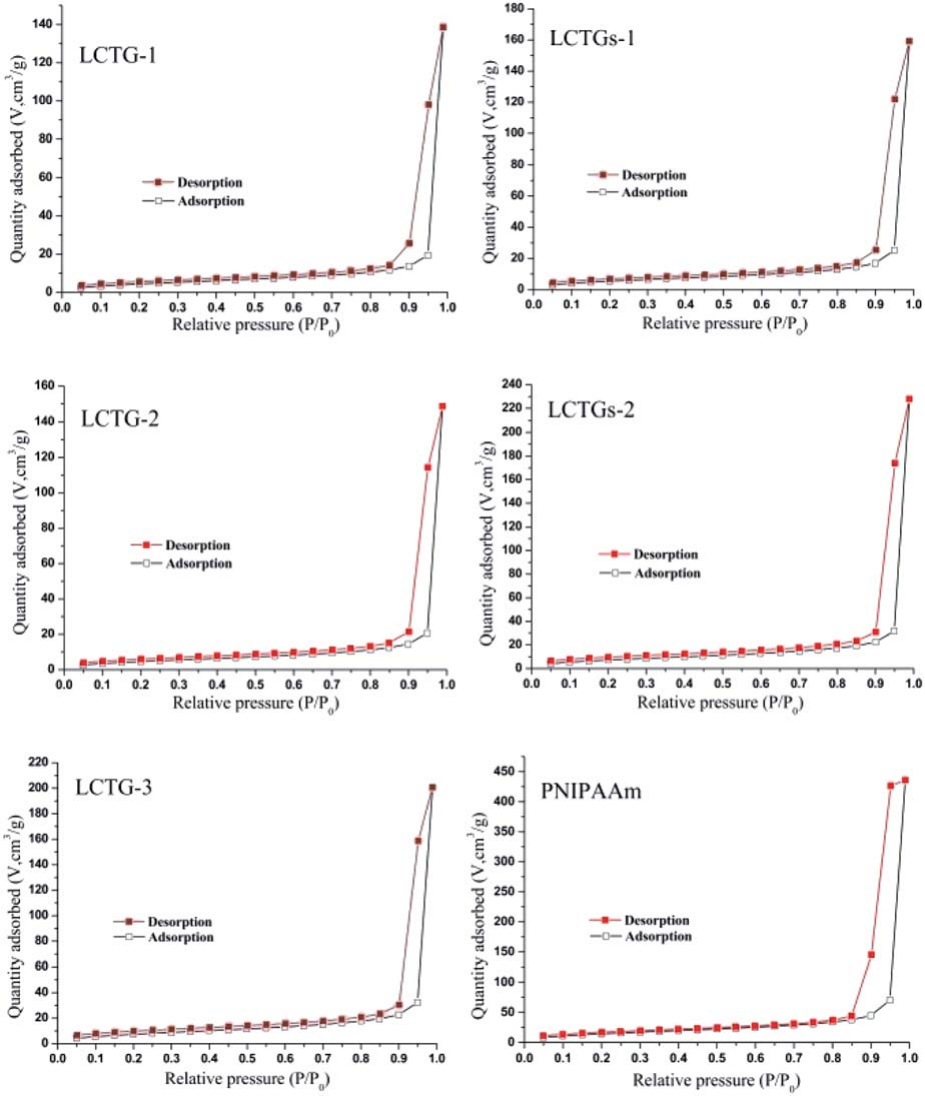
Nitrogen adsorption and desorption isotherms of the SIPN hydrogels and PNIPAAm hydrogel.

The nitrogen adsorption–desorption isotherms of these hydrogels are shown in Fig. 4. All the SIPN hydrogels presented typical IV nitrogen adsorption–desorption isotherms.^39^ The appearance of the hysteresis loop indicated multilayer adsorption on the mesoporous material,^40^ and the prominent adsorption at high relative pressures (*P*/*P*_0_) is associated with capillary condensation during multilayer adsorption on the mesoporous material.

As shown in Table 2, PNIPAAm showed the minimum *d*_v_ (17.49 nm) among all the hydrogels, and the *d*_v_ values showed an obvious increase in the SIPN hydrogels. Because the presence of lignocellulose during the synthesis procedure of the SIPN gel could restrict the reaction between the monomer and the crosslinker, the cross-linking ratios were decreased and induced more pores with larger sizes; this finally resulted in larger *d*_v_ values than that of the PNIPAAm hydrogel. LCTG-1 shows the minimum *S*_BET_ (17.6 m^2^ g^−1^) and the maximum *d*_v_ (31.19 nm) among the lignocellulose SIPN samples. This may be caused by the existence of native lignin, which restricted the dissociation of cellulose and hemicellulose and then decreased the interlacing of PNIPAAm and lignocellulose. As the lignin content decreases, LCTG-2 and LCTG-3, which contain lignocellulose with lower native lignin contents, present greater *S*_BET_ and lower *d*_v_ values than LCTG-1; especially, LCTG-3 gave the greatest *S*_BET_ (29.2 m^2^ g^−1^).

**Table 2:** BET surface areas (*S*_BET_) and BJH average pore diameters (*d*_v_) of the hydrogels

Sample	*S*_BET_ (m^2^ g^−1^)	*d*_v_ (nm)
LCTG-1	17.6	31.19
LCTG-2	18.3	31.07
LCTG-3	29.2	30.92
LCTGs-1	21.7	31.05
LCTGs-2	28.4	30.96
PNIPAAm	57.2	17.49

For LCTGs-1 and LCTGs-2, the dissolved soda lignin in LiCl/DMSO also restricted the reaction between the monomer and crosslinker, then induced more large pores in the gel network; this resulted in larger *d*_v_ and lower *S*_BET_ values in LCTGs-1 and LCTGs-2 compared with LCTG-3, which originated from the same LC-3. Also, *S*_BET_ decreased and *d*_v_ increased with increasing externally added lignin content. Furthermore, both of these hydrogels presented relatively higher *S*_BET_ and smaller *d*_v_ values than LCTG-1 and LCTG-2, which have the same respective lignin contents.

### LCST of SIPN hydrogels

**Fig. 5:**
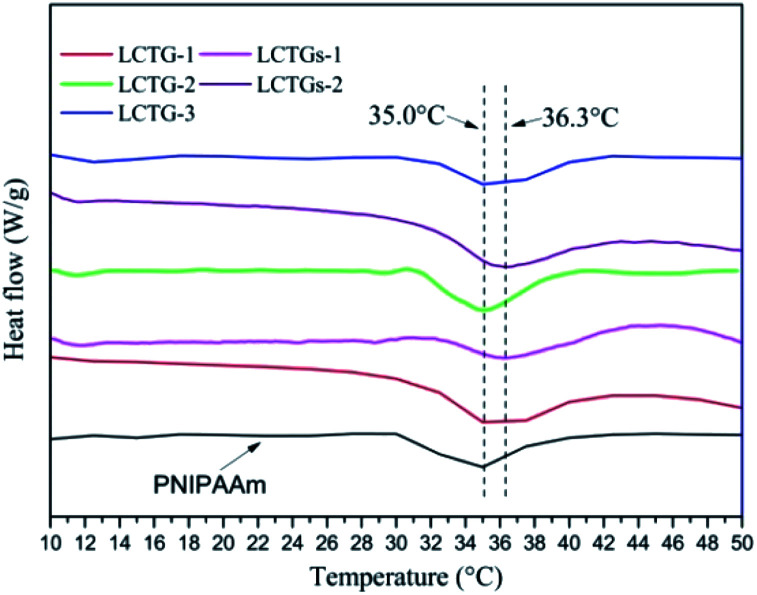
DSC curves of the SIPN hydrogels and PNIPAAm hydrogel.

The DSC curves of the SIPN hydrogels and PNIPAAm hydrogel are shown in Fig. 5. Due to the existence of a hydrophilic/hydrophobic balance in the PNIPAAm network, these SIPN hydrogels can be sensitive to temperature variations of the external environment. If the environmental temperature is lower than the LCST, the hydrophilic groups in the PNIPAAm chains play the main role and interact with the water molecules through hydrogen bonding; then, the hydrogels are swelled by water. Meanwhile, if the environmental temperature is higher than the LCST, the carbonyl groups combine with amino groups to form intramolecular hydrogen bonds which lead to a compact PNIPAAm network; then, the hydrophilic groups are surrounded by the hydrophobic isopropyl groups, and the hydrophobic groups become the prominent part.^41–43^ As a result, the hydrogen bonding between the PNIPAAm chains and water is decreased, the hydrogels shrink, and the absorbed water is expelled.

LCTG-1, LCTG-2 and LCTG-3, which contain different amounts of native lignin, have the same LCST of 35 °C. These data are similar to that of the PNIPAAm hydrogel. As a result, the introduction of lignocellulose with native lignin into the PNIPAAm network through the SIPN strategy will not break the hydrophilic/hydrophobic balance. However, the composite hydrogels LCTGs-1 and LCTGs-2, which contain some amounts of externally added free lignin particles, showed LCSTs 1.3 °C higher than that of the PNIPAAm hydrogel in this system. In the networks of the composite hydrogels, the externally added lignin particles deposited on the lignocellulose chains and PNIPAAm chains by forming van der Waals forces and intermolecular hydrogen bonds with lignocellulose and PNIPAAm. This increased intermolecular hydrogen bonding can improve hydrophilic interactions and partially alter the competition between inter- and intra-molecular hydrogen bonding interactions in the PNIPAAm system; also, the lignin particles can partially prevent interactions of the hydrophobic groups in the PNIPAAm network, consequently increasing the LCST to 36.3 °C.^44,45^

### Mechanical properties of the SIPN hydrogels

**Fig. 6:**
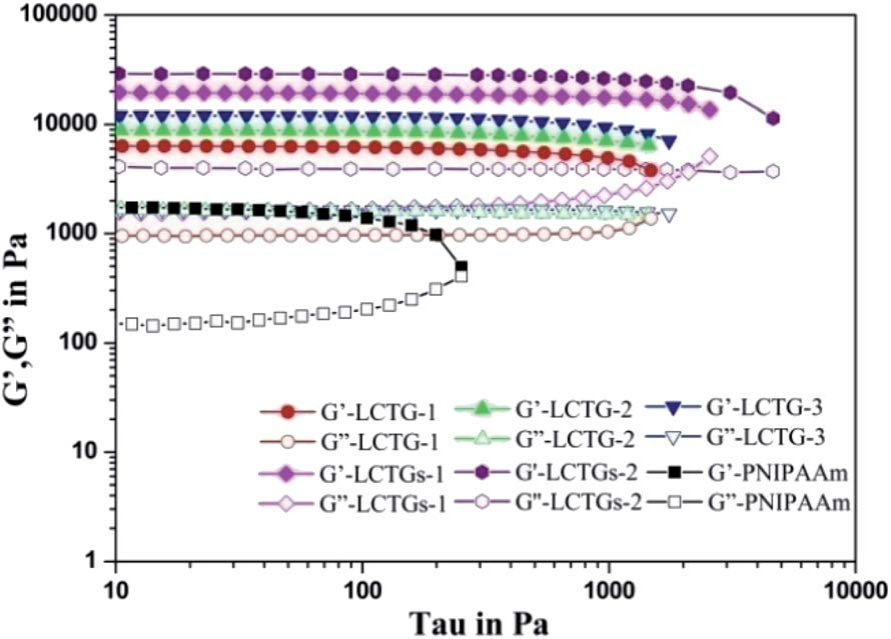
Stress sweeps of the SIPN hydrogels and PNIPAAm hydrogel.

**Fig. 7:**
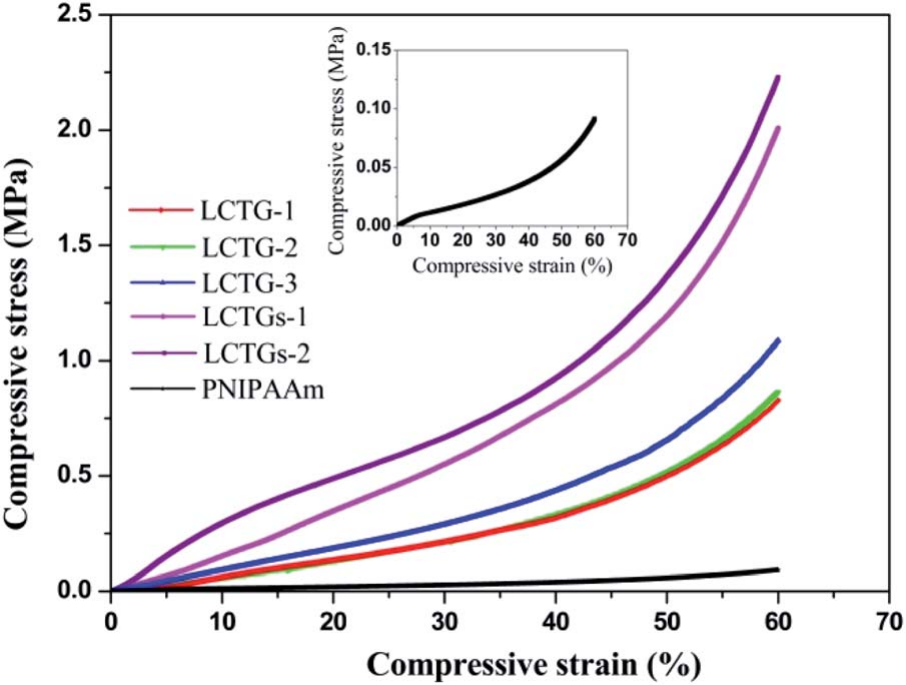
Compression stress–strain curves of the hydrogels.

The influence of lignin on the mechanical properties of the hydrogels is presented in Fig. 6 and 7, which show the corresponding viscoelastic and compressive properties. The storage modulus (*G*′) and loss modulus (*G*′′) in stress sweeps of the SIPN hydrogels correspond to the elasticity and viscosity of the materials, respectively. As shown in Fig. 6, the *G*′ values of all the samples are much higher than the *G*′′ values, and the *G*′ values of the SIPN hydrogels are much higher than that of the PNIPAAm hydrogel. This proves that all the hydrogels are typical elastic gels^46,47^ and that the introduction of lignocellulose into the PNIPAAm network can effectively improve the viscoelasticity of the hydrogels. This can be attributed to the physical entanglement of poly-NIPAAm and lignocellulose, which extended as rigid rods and structural supports in the SIPN hydrogel network.^44^

As the oxygen delignification increased, *G*′ of LCTG-1, LCTG-2 and LCTG-3 gradually increased from 6344 to 12 018 Pa. This indicates that delignification is beneficial to improve the viscoelastic properties of the hydrogels. Because the presence of native lignin could restrict the dissociation of cellulose and hemicellulose, the aggregation of these three components limited the crosslinking of the PNIPAAm network. Thus, the resulting loose structures and large pore sizes decreased the *G*′ value. However, for the composite SIPN hydrogels LCTGs-1 and LCTGs-2, the values of *G*′ are 19 515 and 28 997 Pa. These were much higher than that of LCTG-3. In the composite SIPN hydrogel network, the embedded lignin particles acted as spacers and restricted the movement of the polymer chains, which induced the increase of the storage modulus (*G*′). This proves that the external addition of soda lignin is helpful to improve the viscoelasticity of the hydrogels.

The compressive strength of a hydrogel is an important property for many actual applications. Also, it represents the ability of the hydrogel to resist the external load under compression. The compressive stress–strain curves of the fabricated hydrogels are presented in Fig. 7. It can be observed that introduction of lignocellulose into the PNIPAAm network could also lead to higher compressive stress. At 60% strain, the compressive stress of LCTGs-2 was as high as 2.2 MPa, 21 times higher than that of the PNIPAAm hydrogel. For LCTG-1, LCTG-2 and LCTG-3, the compressive stresses at 60% strain increased with decreasing lignin content. As the oxygen delignification increased, cellulose and hemicellulose could be dissociated gradually with the removal of lignin, and the restriction between the monomers and crosslinker was decreased. Thus, LCTG-3 with lower lignin content possessed a relatively denser porous morphology, leading to higher compressive strength. Meanwhile, for the composite SIPN hydrogels, the stresses of LCTGs-1 and LCTGs-2 at 60% strain were 2.0 and 2.2 MPa, which were higher than that of LCTG-3. As indicated above, the externally added lignin dispersed in the SIPN network and acted as spacers which could facilitate the transfer of the compressive stress, thus enhancing the resistance to external compression. These results prove that the external addition of soda lignin is beneficial to improve the mechanical strength of the hydrogels.

### Reswelling of SIPN hydrogels

**Fig. 8:**
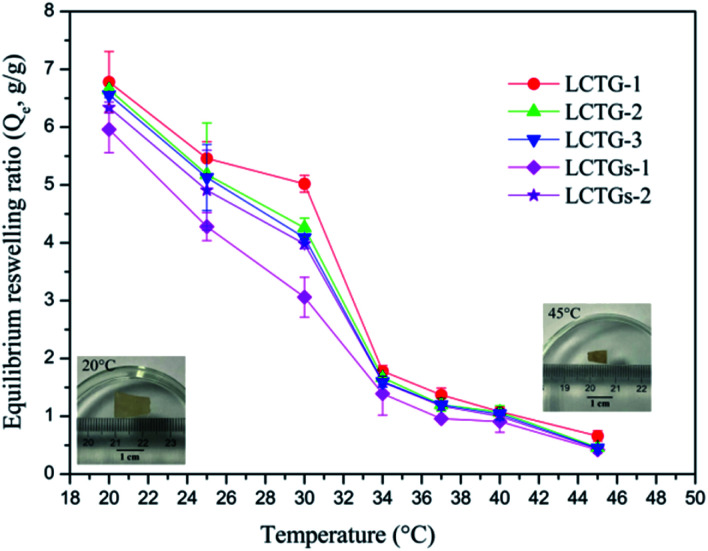
Equilibrium reswelling ratios of the hydrogels at different temperatures and images of an SIPN hydrogel (LCTG-3) at 20 °C and 45 °C after equilibrium reswelling in deionized water.

The equilibrium reswelling ratios (*Q*_e_) of the SIPN hydrogels in deionized water at different temperatures are shown in Fig. 8. It was observed that all the SIPN hydrogels exhibited classical thermosensitivity. As the temperature increased from 20 °C to 45 °C, the *Q*_e_ showed a significant decrease. The obvious dimensional changes of the samples in the inserted images further indicated their thermoresponsiveness. Generally, if the temperature is below the LCST, the whole hydrogel network is well swollen due to the hydrogen bonding between hydrophilic groups and water. However, if the temperature becomes higher than the LCST, the hydrophobicity of the polymer chains is stronger than their hydrophilicity,^41,42,48^ and the reswelling ratios decrease accordingly.

For LCTG-1, LCTG-2 and LCTG-3, the *Q*_e_ decreased with increasing oxygen delignification, which could decrease the native lignin content in the hydrogels. Combining the nitrogen adsorption analysis of these three hydrogels, their *d*_v_ values decreased and their *S*_BET_ values gradually improved with decreasing lignin content. All these results suggest that the presence of native lignin, which restricted the dissociation of cellulose and hemicellulose, may be helpful to form pores with relatively large sizes; these large pores are conducive to improving the corresponding water uptake capacity and to obtaining better *Q*_e_.^45^ However, for LCTGs-1 and LCTGs-2, both their *Q*_e_ values are slightly lower than that of LCTG-3, which contains the minimum amount of lignin. In the networks of composite hydrogels LCTGs-1 and LCTGs-2, the externally added lignin particles were deposited on the lignocellulose and PNIPAAm chains or dispersed within the hydrogel network. This could partially restrict the extension of the hydrated polymer chains when the hydrogels were swelled in distilled water. Additionally, these lignin particles are hydrophobic; they could also limit the water absorption capacities of the hydrogels, especially in LCTGs-1, which contains the maximum amount of externally added lignin.

**Fig. 9:**
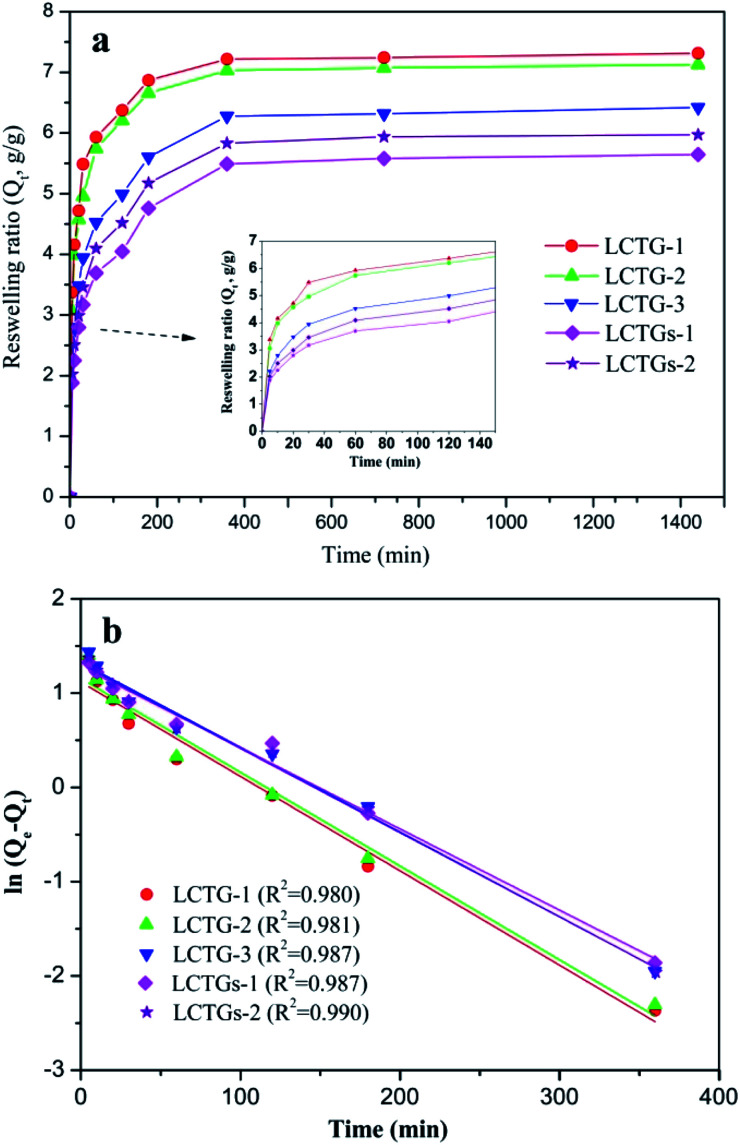
Water absorption kinetics of SIPN hydrogels at 20 °C (a) and pseudo-first-order kinetic model plots (b).

Fig. 9a displays the water absorption kinetics of the SIPN gels at 20 °C. It can be observed that all the samples could reach equilibrium swelling after 6 h. Also, LCTG-1 showed a faster reswelling rate than LCTG-2 or LCTG-3. Generally, there are three steps for hydrogel reswelling upon immersion in distilled water: diffusion of water into the polymer network, relaxation of hydrated polymer chains and subsequent expansion of the polymer network into the distilled water. Therefore, the interior morphology of the hydrogels is important to their reswelling rate. For lignocellulose SIPN hydrogels LCTG-1, LCTG-2 and LCTG-3, the decreased pore size could restrict the diffusion of water into the network and the extension of the hydrated polymer chains, resulting in a decrease of the reswelling rate from LCTG-1 to LCTG-3. Meanwhile, for LCTGs-1 and LCTGs-2, the externally added lignin particles deposited on the lignocellulose chains and PNIPAAm chains could partially restrict the extension of the hydrated polymer chains, resulting in a decrease of the reswelling rate.

The water absorption kinetics were evaluated with the pseudo-first-order model,^49^ which is expressed as:

3 ln(*Q*_e_ − *Q*_*t*_) = ln *Q*_e_ − *kt*

where *Q*_e_ and *Q*_*t*_ are the water absorption capacities (g g^−1^) at equilibrium swelling and at a given time *t* (min), respectively, and *k* is the swelling kinetic constant.

The swelling kinetic constant *k* was determined from the slope of the plot of ln(*Q*_e_ – *Q*_*t*_) *versus t*, as shown in Fig. 9b. It can be seen that the water absorption process basically obeys first-order absorption kinetics. Also, the *k* values of LCTG-1, LCTG-2, LCTG-3, LCTGs-1 and LCTGs-2 are 0.0100, 0.0098, 0.0091, 0.0086 and 0.0089, respectively; this indicates that LCTG-1, which has relatively large pore sizes, has the highest water absorption speed at 20 °C.

### Oil absorbency of the SIPN gels at 45 °C

**Fig. 10:**
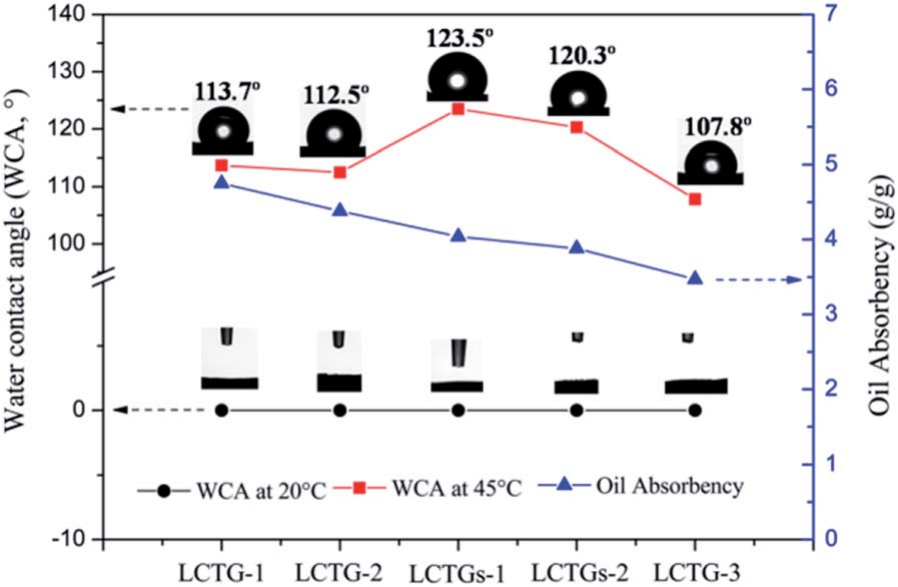
Water contact angles (WCA) of the SIPN gels at 45 °C in air and oil absorbencies of the SIPN gels at 45 °C.

The hydrophobic properties of the SIPN gels at 45 °C were further demonstrated by their water contact angles (WCA). As shown in Fig. 10, all the SIPN gels exhibited rapid conversion from hydrophilicity (WCA = 0°) at 20 °C to hydrophobicity (WCA > 90°) at 45 °C. Also, the WCA values of the samples increased with increasing lignin content. Meanwhile, on the basis of the above work, the as-prepared SIPN gels were employed to absorb soybean oil from oily water at 45 °C, as shown in Fig. 10. When immersed in the oil/water mixture at a higher temperature, the intramolecular hydrogen bonds that formed between the C=O groups and N–H groups led to a compact PNIPAAm network; thus, the hydrophilic groups were surrounded by isopropyl groups.^43^ Then, the hydrophobic groups remaining on the surface of the skeleton played the leading role, resulting in hydrophobic and oil-absorbing gels with potential applications for oil/water separation.

In Fig. 10, LCTG-1 shows the highest oil absorbency among these samples at 45 °C. Considering the SEM and BET results, the presence of native lignin, which restricted the dissociation of cellulose and hemicellulose, may be helpful to form pores with relatively large sizes; these large pores inside the gels endow them with better oil absorption performance at 45 °C, which is above their LCST. As a result, for LCTG-2 and LCTG-3, the oil absorbency decreased with decreasing lignin content. For the same reason, the oil absorbencies of LCTGs-1 and LCTGs-2 were slightly higher than that of LCTG-3. Additionally, lignin is a hydrophobic component, and its presence may also lead to higher hydrophobic character and improve the oil absorption performance.

## Conclusions

A series of thermo-sensitive lignocellulose hydrogels were fabricated with NIPAAm through the SIPN strategy. All the SIPN hydrogels exhibited more favorable mechanical properties than the signal PNIPAAm network due to their physical entanglement, and the externally added lignin in the composite hydrogels was beneficial to obtain better mechanical strength. Also, the lignin present in the hydrogel network resulted in the formation of large pores, and it may be possible to tailor the mechanical properties and morphologies of the hydrogels by varying the existential state and content of lignin. Furthermore, the resulting SIPN hydrogels exhibited classical thermosensitivity, showing good switching between hydrophilicity and hydrophobicity below and above the LCST. Also, the presence of lignin could further improve the hydrophobicity of the gels. With this special switching, the SIPN gels could be used to absorb oil from an oil/water mixture at 45 °C, showing potential applications in oil/water separation.

## Conflicts of interest

There are no conflicts to declare.

## Supplementary Material
